# German Pantheism: The Theory of Life

**Published:** 1849-01-01

**Authors:** 


Art. IV.—*General Principles of the Philosophy of Nature, with an Outline of some of its recent developments among the Germans; embracing the Philosophical Systems of Schelling and Hegel, and Oken's System of Nature.* By J. B. Stallo, M.A. London: Chapman. 1848.

*The Idea of Life. Hints towards the Formation of a more perfect Theory of Life.* By S. T. Coleridge. Edited by Seth B. Watson, M.D. London: Churchill. 1848.

The history of speculative philosophy, whether ancient or modern, enunciates in language sufficiently articulate the inability of man to grapple with the mysteries of the spiritual world. He may analyze rigorously his inward thoughts and feelings, or curiously explore the occult qualities ascribed to matter; he may speculate on the nature and destiny of the human mind, and the manifestations and existence of the Deity, as revealed to him in the harmonies and beauties of the surrounding universe; but as he progresses in his investigations, and dives deeper and deeper into the secrets he seeks to penetrate, he finds that all his efforts and ingenuity recoil upon him—"thus far shalt thou go, and no farther," seems to resound upon his ear as from the voice of the Great Unknown, and he stands, with his pretensions humbled, upon the shore of humanity, gazing, conscious of his weakness, upon a wide unfathomable sea—a finite being, vainly endeavouring to compass and comprehend that which is Infinite, and overstep the insurmountable boundary which divides the Creator from the created. The futility of all such attempts is notorious; yet there have existed master spirits in the regions of philosophy—minds so highly gifted as to perceive the relations of the most intricate phenomena, and detect the laws by which they are governed. The history of the human intellect shows that men occasionally arise whose perceptions intuitively extend beyond the knowledge of their age. They are the prophets and seers of the olden time. They make discoveries—they point out facts—they lay down principles which become as beacon-lights for the guidance of after generations. Such men were Plato, Aristotle, Bacon, Kepler, Sir Isaac Newton, and many other illustrious philosophers who have accomplished their high mission, and contributed to the intellectual progression of mankind, and the civilization and refinement of nations. For although, in the first instance, the truths of science may not be immediately impressed upon the conviction of the multitude, yet it is the privilege of the higher order of mind to extend its influence by degrees downwards upon the lower order of mind, until the mass of mankind imperceptibly becomes actuated by every new discovery which marks the intellectual character of the age. "If we look for a moment," says Morell, "at the law by which thought is propagated, we find that it always descends from the highest order of thinkers to those who are one degree below them; from these again it descends another degree, losing at each step of the descent something more of the scientific form, until it reaches the mass in the shape of some admitted fact —a fact which rests on the authority of what all the world above them says, and which, therefore, they receive, totally regardless of the method of its elimination."* If this law of the descent of thought, or, as it is otherwise expressed, this "gravitation of ascertained truth from the higher order of minds to the lower,"† produces results which affect so directly the most vital interests of society, it behoves us, in entering into the regions of speculative philosophy, to proceed with caution in every induction we attempt; for out of the shadows of false theories social doctrines as false have not unfrequently been evolved, which have threatened for awhile to spread darkness and confusion over the whole moral world.

The existence of a Deity, the immateriality of the human mind, the independent nature of the vital principle, involve reasonings and deductions which are more closely allied to each other than might at first sight be supposed. "What," says Dr. Barclay, "are the causes of organization, and the other concomitant phenomena of life? Some have imagined that these may originate in certain unknown properties of the atoms of which organized structures are composed. On this hypothesis human existence terminates with death, schemes of expediency and self-interest take the place of religious and moral obligations, and thoughts and actions, however criminal, are, like Spartan thefts, to be held disgraceful only if detected. From the same hypothesis it

* Morell's History of Speculative Philosophy, vol. i. p. 19.

† Ibid. p. 21.

follows, as a corollary, that if atoms can construct a human organism, and exhibit the phenomena of a human soul, they may, when collected in greater numbers, produce a Deity."* These considerations attach a very serious importance to the subjects which we are about to consider. But our fair readers, if any such grace our pages by their perusal, need not be startled at this our prelection; for they may rest assured that the breastplate of truth is proof against any unrighteous weapons of attack. Nay, these subjects, however sacred they may be, invite, or rather seem to challenge, investigation, for our belief in the most fundamental principles of religion must be associated with, and verified by, our observation of the phenomena of the surrounding world, and must be strictly in accordance with those laws which immediately affect the conviction of the human understanding. We may enter, therefore, upon our task with a confiding and fearless spirit, because it is by such means only that we are led to weigh and sift the integral elements of the faith which must constitute the very basis upon which every moral and religious system is eventually raised. To a philosophical mind, such can never, although holy, be forbidden ground, notwithstanding the many intricate and perplexing paths which open around us.

These preliminary observations have been suggested by the two volumes before us—"*The Philosophy of Nature*," by J. B. Stallo, (a professor of analytical mathematics in the United States,) and "*The Idea of Life*," by Samuel Taylor Coleridge. The soil of Germany has always been prolific in mystery; it is the very Brocken of the intellectual world. To follow the phantasies which rise out of the arcana of transcendentalism requires no ordinary perseverance; and, after all, we fear that it is a fatiguing and unprofitable task. The language we meet with is exoteric, and in the first instance bewildering; and when we have grappled with some vague and shadowy proposition, which threatened to elude our vigilance, and have resolutely succeeded in tearing it from "its shady den and its bristling entrenchments of uncouth terminology," we not unfrequently, as Carlyle truly observes, find that we have, after all, only mastered some "very harmless truth, familiar to us from old acquaintance—so familiar as to be a truism; and too often we are reminded of Dryden in the Battle of the Books; there is a helmet of rusty iron—dark, grim, gigantic—and within it, at the farthest corner, a head no bigger than a walnut."† We are informed that the Egyptian priests were wont to hide, under the veil of symbols, truths which they deemed too sacred to be contemplated by ordinary mortals; and, in imitation of them, we presume, the philosophers of Germany enshroud their meanings, or rather their no meanings, in studious linguistic obscurity, which

* Barclay on Life and Organization, p. vii., Preface.

† The Life of Frederick Schiller, p. 175.

leaves us to grope our way in the dark, or deal with vain shadows. Hence, no sooner have we opened these mystic pages, than our trans-atlantic Mephistopheles transports us at once into the very "thick of the witch element." Amidst the abstruse and mystical categories of Kant, the *egoism* and *non-egoism* of Fichte, the absolutism of Schelling, and the pantheism of Hegel, we may well call out, like Faustus in the Wal-purgen, for some "zig-zag light" to guide us on our way—

> "The limits of the sphere of dream,
> The bounds of true and false are past,
> Lead us on, thou wandering gleam!" *

But before we permit ourselves to be winged away into these twilight realms of imagination, let us pause. "*Les Allemands mêlent ils trop souvent la métaphysique à la poesie,*" observes Madame de Staël; and certainly we do find that most of their poets are philosophers, and most of their philosophers poets. "Nothing," observes Mr. Hume, "is more dangerous to reason than the flights of imagination, and nothing has been the occasion of more mistakes among philosophers. Men of bright fancies may in this respect be compared to those angels which the Scripture represents as covering their eyes with their wings."†

But the German mind is imbued with a peculiar disposition to indulge in mysticism and idealism; hence the spirit of pantheism will be found to pervade, more or less, every system of German philosophy. The fundamental principle of this doctrine is, that the material world, in all its varied phases, is a development from, or an extension only, or self-revelation, of the Creator. This attempt at the *deification* of the material and spiritual phenomena of the universe, as Schlegel designates it, carries us back to the system of emanation, commonly embraced by the ancient barbaric philosophers, and afterwards admitted into the early theogonies of the Greeks.‡ It was affirmed by Plato, that the soul of man and the essential forms of things proceeded by emanation from the Deity.§ The same doctrine, under certain modifications, was adopted by Aristotle.§ Pythagoras taught that God was the universal mind diffused through all things, and the source of all animal life.‖ In the first centuries of the Christian era, the Cabbala (a pretended illumination invented by the Rabbi Akibha and his disciple Simeon Ben Jochai, the spark of Moses which the Jews affect to have received through tradition from a Divine source) set forth, that all things that exist are penetrated with and partake of the Divine nature.¶

* Shelley's translation of The May-Day Night.
† Treatise of Human Nature, vol. i. p. 464.
‡ Enfield's History of Philosophy, from Brucker, vol. i. pp. 54, 330.
§ Ibid. pp. 238, 278. ‖ Ibid. p. 393.
¶ Ibid. vol. ii. p. 267. See also Tennemann's History of Philosophy, p. 182.

To this theory of emanations was afterwards attached a tissue of imaginations, describing the counteracting influence of demons, which gave rise to the belief in magic, demoniacal possession, and subsequently witchcraft, the pseudo principles of which imposed for many ages upon the credulity of mankind, and really produced, in many well-attested cases, physiological effects, very identical with the phenomena now ascribed to animal magnetism.*

Hence pantheism has, under various modifications, been transmitted from the earliest ages through successive systems of philosophy; but Germany, above all European countries, has both in feeling and speculation constantly reverted to it. Her poets, her artists, her musicians, have been all more or less pantheists; the principles of pantheism are as clearly developed in the effusions of Goethe as in the mystical writings of Novalis.† "In the floods of life," says Goethe, "in the storm of deeds, I move up and down, I weave to and fro! Birth and the grave an eternal sea, a changing strife, a glowing life! Thus I create at the roaring loom of Time, and weave the living garment of the Deity."‡ But the pantheism of poetry should, we venture to suggest, be distinguished from the pantheism of philosophy. The one is a deeply felt recognition of an all-pervading Divine agency, revealing itself through the universality of nature: the other is an attempt, whether by emanation, evolution, or extension, to account for the same Divine manifestations by identifying the great First Cause with the phenomena themselves. The one uplifts its orisons in acknowledgment of the omnipresence of a Supreme Being, expressing in its adoration

"A sense sublime
Of something far more deeply interfused;
Whose dwelling is the light of setting suns,
And the round ocean, and the living air,
And the blue sky, and in the mind of man,
A motion and a spirit that impels
All thinking things, all objects of all thoughts,
And rolls through all things." §

---

* On this curious subject see an article in the Polytechnic Magazine; page 86, by Thomas Stone, M.D. Mortimer, 1844.

† Lewe's Biographical History of Philosophy, vol. iv. p. 184.

‡ In Lebensfluthen in Thatensturm
Wall' ich auf und ab
Webe hin und her!
Geburt und Grab
Ein ewiges Meer
Ein wechselnd Streben
Ein glühend Leben!
So schaff 'ich am sausenden Webstuhl der Zeit,
Und wirke der Gottheit lebendiges Kleid."

§ Wordsworth; Lines on Tintern Abbey.

The other, instead of enlarging our sense of the Divine Attributes, annihilates them, by destroying the personality of the Deity. The pantheism, however, which sprung out of the ancient systems of cosmogony was avowedly an idealism only, a pure fiction of the imagination; for although the dialectic form of reasoning prevailed in the schools of philosophy, the pantheistic principles were not founded upon any kind of induction until after the revival of letters, when Spinoza, availing himself of the doctrines of Descartes, founded upon them a system of pantheism, which was so logically derived from the Cartesian premises, that, were these admitted to be correct, his conclusions would even to the present day remain incontrovertible.

The age in which Spinoza lived was essentially a speculative one; the spirit of metaphysical research was abroad; the arena which had been the scene of the bitter controversies and unseemly wranglings of the Schoolmen, was at length occupied by a more temperate class of philosophers. In England, Locke first laid the foundation of what has been since appropriately called the sensational school of philosophy, from the doctrines of which Hume, by a just logical deduction, founded a superstructure of scepticism, which in its turn roused the energies of Reid, and startled Kant from his "dogmatic slumber." So that from the scepticism of Hume, mediately or immediately, as observed by Sir William Hamilton, "all our subsequent philosophy has been evolved; and the doctrines of Kant and Reid," adds Sir William, "are both avowedly recoils from the annihilating scepticism of Hume; both attempts to find for philosophy deeper foundations than those which he had so thoroughly subverted."* Meanwhile, in France, Descartes, who was, by the unfortunate Condorcet, designated "the father of unfettered philosophical inquiry," promulgated his system, fragments of which have been found scattered over every part of the civilized world. "In his own day, and long after his day, he was," observes Blakey, in his recent History of Philosophy, "all-powerful. We find his disciples among persons of all shades of theological belief. In the bosom of the Catholic Church we see Arnauld and Pascal, and Fenelon and Bossuet, and Malebranche embracing and expounding his doctrines with all possible zeal."†

Strange, indeed, that a system which excited so much admiration in minds so orthodox, and produced such an influence throughout Europe, should have given rise, and that by a logical deduction so strictly legitimate as to have "all the appearance of mathematical strictness,"‡ to a pantheistical result, which has been stigmatized, somewhat undeservedly,

* Sir William Hamilton's edition of Reid's works, p. 91.

† Blakey's History of the Philosophy of Mind, vol. ii. p. 233.

‡ Tennemann, op. cit., p. 327.

perhaps, as the worst species of atheism! The philosophy of Spinoza teaches that there exists, *in universum,* but one substance infinite in its nature, and that substance is God. "Whatever exists exists in God; for external to his existence nothing can positively be conceived. All things are but the manifestation of his being, and the whole is bound together by an inexorable necessity." * That Spinoza affirmed the existence of a God, and affirmed it so earnestly as to merit the appellation given to him by Novalis, of "the God-intoxicated man," may readily, observes Morell, "be admitted in a certain sense; but that he allowed the existence of a God, in the ordinary and Christian acceptation of that word, is far from being the case. A being to whom understanding, will, and even *personality,* is denied—a being who does not create, but simply *is*—who does not act, but simply unfolds—who does not purpose, but brings all things to pass by the necessary law of his own existence; such a being cannot be a father, a friend, a benefactor—in a word, cannot be a God to man, for man is but a part of himself."† "It may be more correct to term the philosophy of Spinoza a pantheism than an atheism; but if we take the common idea or definition of deity as valid, then assuredly we must conclude that the God of Spinoza is no God, and that his pantheism is only a more imposing form of atheism.‡"

The Philosophy of Nature, by Stallo, presents us with a pantheism of the same character; after describing which, he gives us a condensed view of the doctrines of Kant, Fichte, Schelling, and Hegel, with the visionary system of nature propounded by Oken. In order to appreciate the fundamental principles of these dreamy theories, we must set out by understanding clearly the meaning which these philosophers attach to the words *subjective* and *objective.* Thus, a man's faculties and acts are attributes of which he is the *subject;* the knowledge, therefore, which belongs to his own mind, and which he derives from his own consciousness, is *subjective:* whereas that which he derives from the observation of the surrounding world, or external *objects,* is *objective.* The one is the inward—the other the outward world of existence; and to establish a clear relation between them has been the difficulty of all philosophy. All our ideas, according to Kant, have a double origin—partly subjective, and partly objective; the mind, he admits, has pure

* Blakey, loc. cit., p. 306.

† Peter Bayle, in refuting the doctrine of Spinoza, ridicules it, by saying that if it were true, in the war raging between the Turks and the Austrians, the Deity was just then cutting himself to pieces. (Art. Spinoza, Dict. Hist. et Critique.) These pseudo-philosophers were wont to take strange liberties: "To-morrow," said Hegel, at the end of one of his lectures, "To-morrow I shall proceed, gentlemen, to create God!".

‡ Morell, op. cit., vol. i. p. 188.

suggestive principles of its own, but also derives some perceptions and sensations from without; and the two co-operate to produce knowledge. On the other hand, Fichte—calling the subjective *ego,* and the objective *non ego*—argued that, although certain mental conceptions were *in me,* they were caused by something *out of me,* so that the *ego* and *non ego* became identical. "The fundamental error of Fichte," says Morell, "was, that he entrenched himself so closely within the circle of his consciousness, that it was impossible for him to find any passage from thence into the surrounding world."* After Fichte, came Schelling—the Plato, as he is sometimes called, of modern Germany—who maintained that the *ego* and *non ego* (subject and object) are identical, and exist only in the absolute—that is to say, the Absolute, by which he means the Deity, contains within itself, potentially, all that it afterwards becomes by its own self-development. This self-development, be it observed, is not the free and designed operation of intelligence, but rather a blind impulse, working unconsciously in nature, and only coming to self-consciousness in mind. On this principle, all difference between God and the universe is completely lost; his pantheism becomes as complete as that of Spinoza; and "as the absolute is supposed to be evolved from its lowest forms to the highest, in accordance with the necessary law or rhythm of its being, the whole world, material and mental, becomes one enormous chain of necessity, to which no idea of free creation can by any possibility be attached."† Lastly, after the philosophy of Schelling had obtained a vast popularity in Germany, came Hegel, who boldly started with the postulate, that "being and non-being are the same;" and, denying the existence of both object and subject, insisted that everything was purely ideal. The only real existence which he admits is one of relation;—"the whole universe is a universe of ideas—a process or movement of the Deity ever unfolding itself, but never unfolded."‡ Such are the pantheistical systems which appear to have fascinated the imagination of Stallo, but which he has explained in a very obscure and imperfect manner. A cloudy element, however, is the most congenial atmosphere for all such fictions; and the unintelligibility of the language in which they are conveyed is their best security against popular ridicule.

"*The Idea of Life, or Hints for the Formation of a more Comprehensive Theory of Life,*" here claims attention; and, assuredly, the very title of this little volume by Samuel Taylor Coleridge, suggests the anticipation of paradoxes as subtile, dreams as wild, and phantasies as startling, as any we have just adverted to. It was observed in the early life

* Morell, op. cit., vol. ii. p. 89. † Ibid. p. 118. ‡ Ibid. p. 137.

of Coleridge, that he was endowed with a peculiar mental idiosyncracy, which predisposed him, even in childhood, to self-meditation and intellectual seclusion. He describes himself as a child, taking his "little stool" and his "little book," and sitting by the side of his mother, and listening to the talk of his elders. "Huffed away from the enjoyment of muscular activity, I was driven," he observes, "from life in motion to life in thought and sensation. . . . Alas! I had all the simplicity, and all the docility of the little child, but none of the child's habits. I never thought as a child, never had the language of a child."* It is well observed by Wordsworth, "the child is father of the man;" and Coleridge, in his after-life, manifested the same disposition to retire intellectually within himself, and dwell within the sphere of his own idealism. He did not tread the common paths of knowledge and philosophy. He stood as much apart, in his philosophical spirit, from other men as Manfred himself, soliloquizing to the elements on the summit of the Jungfrau. He, too, although no misanthropist, like Manfred, poured forth his incantations, and invoked and introduced supernatural imagery in his poetry with a truly marvellous effect. His "Ancient Mariner," his "Christabel," his "Kublah Khan," sufficiently attest his success in this species of intellectual necromancy; and it may readily be conceived, that when a mind so constituted entered into the phantasmal land of German philosophy, it would easily become fascinated by the very mysteries of its transcendentalism. This was eminently the case with Coleridge; but he entered the labyrinth with a firm step, upheld by the sense of a higher philosophy than any which had ever emanated from its school. Accordingly, when he returned to enlighten the world with the knowledge he had acquired, he did not seek to weave a web of metaphysical sophistry around the attributes of the Supreme Being, mystifying at the same time the phenomena and laws of the surrounding universe, until the relation between cause and effect becomes annihilated. He did not put forth paradoxes to impeach the moral and intellectual responsibility of man, reducing him to the condition of an automatic microcosm, and destroying the elements of all moral obligation; but, deeply impressed with the truths of Christianity, he sought to interweave its fundamental principles with the doctrines he inculcated, and introduced for this purpose a new organ of faith, by the super-rational assistance of which he maintains we are alone enabled to carry on these investigations. Hence Coleridge has the merit of being the first in this country who sought to associate these philosophical speculations with the doctrines of Christianity. While we cheerfully and unreservedly concede every honour to the poet-philosopher, we almost regret the publication of "The Idea of Life," because,

* Gilman's Life of Samuel Taylor Coleridge, vol. i. p. 10.

although as a posthumous volume it may be acceptable to us, proceeding as it does from the pen of one whose memory we so highly esteem, still we consider that it is not likely to add to his reputation, for the views which it contains are not characterized by any originality, and almost in every page challenge contradiction.

He thus opens the subject — "When we stand before the bust of John Hunter,"— we quote the first sentence of the Introduction —" or as we enter the magnificent museum furnished by his labours, and pass slowly with meditative observation through this august temple, which the genius of one great man has raised, and dedicated to the wisdom and uniform working of the Creator, we perceive at every step the guidance, we had almost said, the inspiration, of those profound ideas concerning life which dawn upon us, indeed, through his written works—but which he has here presented to us in a more perfect language than that of words—the language of God himself as uttered by nature." After this noble exordium, we are disappointed to find ourselves hurried into a frivolous and profitless discussion upon the definitions of life; and that Coleridge, instead of following the steps of the genius to whose bust he had just made the above invocation, throws himself into the stream of German idealism, adopting, to its extremest extent, the views of Fichte, Schelling, and Hegel, a pantheistical theory of life, opposed to every principle of reason, and common sense. According to this fanciful hypothesis, which is adopted by Stallo, life itself is supposed to originate in the creative energy of nature, and to become diffused through all objects. " Matter exists not in itself, but in virtue of, and in reference to, its inner vitality." (Stallo, page 31.) The properties of matter, " the extension and impenetrability of matter proceed (not from the laws of attraction, but) from the necessary reality of the unital life, the unity or identity of which life is the object of the whole process of its existence." (Stallo, *ib.*) The true centre of gravity of matter, is in its inner life—the self-moving, and the self-sustaining, which is " the vital, substantial principle of all existences." (Stallo, p. 42.)

Dead matter does not exist—death itself is not death ! " The apparent corpse after the death of any organism is instantly subject to Cosmic vitality: when we imagine we behold matter without life, it is only because this life, on account of its generality, is beyond our immediate ken." (Stallo, p. 35.)

It may be remembered, that Diogenes of Apollonia, following the steps of Anaximenes, asked, " What constitutes the air, the origin of things? Clearly," he answered, " its life." The world he fancied a living unity, and he attributed to it a set of respiratory organs, which he fancied he discovered in the stars. As manifestly preposterous are the propositions of these pseudo philosophers.

"The earth," Stallo quotes from Hegel, "is the crystal of life—the first determinate life is the atmosphere; the second phase of determinate terrestrial life is the neutral earth, the sea; the third phase is the continent —the gigantic corpse of a life gone by—the firm crystal of the lunar element." (Stallo, p. 466.) So also Coleridge argues, that the physical properties of matter are vital in essence—describes gravitation as the universal life, and talks of the "*irritability* which metals manifest to galvanism." (Theory of Life, page 39.) How can any rational man deal with such ravings? Nevertheless, we find that this pantheistical theory of life has recently been propounded at some length, by the eminent Dr. Carus. "Comparing animal with planetary life," says he, "we are led to conclude, that as the blood, a homogeneous fluid in continual circulation, is the source in which all forms and reproductions of organism originate, so is water one of the members most important for the life of the earth. This internal life of the fluid becomes indeed more evident when we consider the individual formation of the solid to which it gives birth. The most striking illustration of this, is the process of crystallization, which exhibits a near approach of the inorganic to the organic life; for we cannot deny even to the crystal a certain inward peculiar life, at the moment of its crystallization. The only difference between an organic body and a crystal is, that the life of the latter, the principle of action and reaction, terminates as soon as its formation is accomplished. One might be tempted to say, that the crystal lives only to form itself, for as soon as it is formed it dies, while true organisms, on the contrary, form themselves only in order to live, and it is only when they are perfectly formed that their life is truly and properly evident."*

This very observation points out the distinction between the construction of a crystal and the development of organization—the nonexistence in the one of any sustaining principle of vitality, and the positive evidence in the other, of the presence of a vital power supporting the organism through all the physiological changes which it undergoes. "But what is life?" asks Coleridge. In reply to which, we may ask—"What is light?" "What is heat?" "What is electricity?" "What is magnetism?" Before we can give a definition of either of these principles, its nature must be fairly revealed to us, because no definition can be satisfactory that is not a clear expression of truth; hence writers on logic, in the middle ages, made definition the last stage in the progress of knowledge. "It is absolutely necessary," observes Whewell, "to every advance in our knowledge, that those by whom such advances are made, should possess clearly the conceptions which they employ;

* Dr. Carus on the Kingdoms of Nature; their Life and Affinity, in Taylor's Collection of Scientific Memoirs. Vol. i. p. 230. 1837.

but it is by no means necessary, that they should unfold these conceptions in the words of a formal definition."* Galileo, Huyghens, Newton, Fresnel, Young, had clear conceptions of the course adopted in the progress of their discoveries; but not one of them could anticipate by a definition the result of their investigations. And so it is with respect to life, as well as light, heat, electricity, and magnetism, neither can be defined with any degree of satisfaction; but the phenomena ascribed to each are not the less legitimate subjects for philosophical research. The definition which Coleridge himself gives of life, after deprecating that of Bichat, (" c'est l'ensemble des fonctions qui resistent à la mort,") is more objectionable than any with which we are acquainted. " I define life as the principle of *individuation*, or the power which unites a given *all* into a *whole*, that is presupposed by all its parts." (Theory, page 42.) A block of granite may be said to have its component parts held together by a " principle of individuation," which " unites the given all" into a mass; but as Coleridge disclaims the division of all that surrounds us into things with life, and things without life; and as he contends that the term life is no less applicable to the irreducible bases of chemistry—such as sodium, potassium, &c.—or to the various forms of crystals, or the geological strata which compose the crust of the globe, than it is to the human body itself; so we (Theory, Preface, page 8) cannot expect that any definition of life from his pen could be acceptable, involving, as it must, such an extravagant hypothesis. We are glad, however, to find that Coleridge repels with so much earnestness the assumption that life is a result of organization — a doctrine we regret to find advocated recently, with much plausibility, by Dr. Carpenter, whose " Manual of Physiology" is at present esteemed (deservedly in other respects) one of the best text-books of physiology in our metropolitan schools of medicine. " I disclaim," says Coleridge, " the error of Stahl in deriving the phenomena of life from the unconscious actions of the rational soul. I repel with still greater earnestness, the assertion, and even the supposition, that the functions are the offsprings of the structure and life the result of organization connected with it, as effect with cause. Nay, the position seems to me little less strange than as if a man should say, that building, with all the included handicraft of plastering, sawing, planing, &c., were the offspring of the house, and the mason and carpenter were the result of a suite of chambers, with the passages and staircases that lead to them. To make A the offspring of B, when the very existence of B, as B pre-supposes the existence of A, is preposterous in the literal sense of the word, and a consummate instance of the *hysteron proteron* in logic. But if I reject the organ as the cause

* Whewell's Philosophy of the Inductive Sciences, vol. ii. p. 14.

of that of which it is the organ, though I admit it among the conditions of its actual functions, for the same reason I must reject fluids and ethers of all kinds, magnetical, electrical, and universal, to whatever quintessential thinness triple distilled and (as it were) super-substantiated. With these, likewise, I abjure all chemical agencies, compositions, and decompositions, they suppose a stimulability, *sui generis,* which is but another paraphrase for life. . . . To account for life is one thing, to explain life is another. In the first we are supposed to state something prior (if not in time, yet in the order of nature) to the thing accounted for as the ground or cause of that thing, or (which comprises the meaning and force of both words as its sufficient cause,) *quæ et facit et subest;* and to this, in the question of life, I know no possible answer but God. To account for a thing is to see into the principle of its possibility, and from that principle to evolve its being; thus the mathematician demonstrates the truths of geometry by constructing them. . . The reasoner who assigns structure or organization as the antecedent of life, who names the former a cause, and the latter its effect, he it is who pretends to account for life. Now Euclid would, with great right, demand of such a philosopher to *make* life,—in the same sense, I mean, in which Euclid makes an icosaedron, or a figure of twenty sides—namely, in the understanding, or by an intellectual construction,—an argument which, of itself, is sufficient to prove the untenable nature of materialism." (Theory of Life, pp. 34, 35, 36.)

All this is well and forcibly argued, yet we cannot but think that before forming, or attempting to form, any conception of the nature of life, it is necessary to observe its development closely in all stages of organization, and the circumstances under which it is preserved or extinguished. The separation between living and inert matter—between organized and unorganized beings, we conceive to be perfectly clear and positive, and we hold that life itself exists only in the vegetable and animal creation. The simplest conditions of organization under which it becomes manifest, are in those cellular, flowerless plants, which are nourished through their whole surface by the medium in which they vegetate, *algæ,* living in water and damp places. Here the question suggests itself, Is the life of plants identical in itself with the life of animals? It is certain that some ambiguous marine productions which Pallas considered to be plants, were afterwards by Lamarck pronounced to be zoophytes, and again, by Kützing and Decaisne, shown to be sea vegetables, a fact which only proves the inaccuracy of observation, and which by no means demonstrates a direct algal alliance between the two kingdoms. It was long ago asserted by Bory de St. Vincent, that there exists in nature organized bodies, which are animal at one period of their lives, and vegetable at another. "Those who have ever examined"

(observes Professor Lindley) "the surface of stones constantly moistened by water, the glass of hot-houses, the face of rocks in the sea, or of walls where the sun never shines, or the hard paths in damp parts of gardens, after rain, cannot fail to have remarked a green mucous slime with which such places are covered; and which consists of algals in their simplest state of organization." "This slime" (says Bory de St. Vincent) "resembles a layer of albumen spread with a brush; it exfoliates in drying, and finally becomes visible by the manner in which it colours green or deep brown. One might call it a provisional creation, waiting to be organized, and then assuming different forms, according to the nature of the corpuscules which penetrate it or develop among it. It may further be said to be the origin of two very distinct existences, the one certainly animal, the other purely vegetable.* Here, it will be observed, that Bory de St. Vincent recognises, in these minute particles, "two very distinct existences;" nor does he adduce any evidence to show that the vegetable corpuscules give rise to the animal development, or that the animal corpuscules give rise to the vegetable development of organization. A curious observation has been made respecting the motion of some minute species of plants—many of the conferva tribe (especially of the genera conferva ulvas and their near allies) produce, in their tubular threads, reproductive bodies or spores, which, after a time, acquire a power of rapid and quasi-voluntary motion while in the inside of their mother; by degrees, and in consequence of their tapping against the soft side of the cell that holds them, they escape into the water; when there they swim about actively, just like animalculi, and at last, retreating to a shady place, attach themselves to a stone or some other body, lose their locomotion, and thenceforward germinate and grow like plants.† This, when we reflect upon it, is not more marvellous than the dispersion of the reproductive seeds of vegetables by the wind upon land, which float hither and thither, with the "down upon the thistle's beard," until they find a congenial soil upon which they fix their habitat. Observation teaches us that all nature teems with life—the air we breathe, the dust upon which we tread, the water with which we slake our thirst, we find, when microscopically examined, loaded with animalculi, and germs, and ova, which rapidly develop into minute beings, curiously organized, and which apparently spring spontaneously into existence. The fact, however, of spontaneous generation has not yet been proved; and in those experiments which pretend to exhibit the development of animalculi in solutions subjected to electrical action, there can be no doubt but their ova pre-existed in the fluid. "In my observations," says Ehrenberg, who may be esteemed the

* Lindley's Vegetable Kingdom, vol. i. pp. 14, 15, *et seq.*

† Ibid.

highest authority on this subject, "pursued with so much zeal for twelve years, I *never* witnessed the spontaneous origin of *one* infusorium from slime or vegetable tissue; and, supported by such experience, I am of opinion, that these animals are never formed by '*generatio primitiva*,' but originate from eggs. The active motions and contractions in plants and their parts, especially *algæ*, ought not to give rise to the supposition of an animal nature, even when they are called infusorial, or animal motions. Internal nutritive organs, and a definite oval aperture for the reception of solid substances, which may be demonstrated, distinguish the apparently most simple animals from plants. I have never seen, in my numerous experiments, the motive algæ seeds take up the smallest quantity of solid nutriment; and thus the fruit-strewing algæ may be distinguished from the monads which swarm around it, in the same manner as the tree from a bird."*

When we leave the microscopic world, and approach other conditions of organization which exhibit little or no complexity of structure—as, for example, when we examine an hydatid or a polypus, or the gelatinous-looking body of the star-fish or the sea-urchin—we again recognise the existence of life in very simple structures—structures so simple as to militate against the idea of life being the result of any complex mechanical or chemical process. In animals, however, which are more highly organized—which possess a complicated nervous, circulating, respiratory, and digestive apparatus, the functions of which are implicated with and modified by certain chemical changes that occur in the ingesta and egesta, and also in the solids and fluids of the system itself—the sources whence life might have originated may, so to speak, appear theoretically multiplied. An infinitely more complicated organization is placed before the theorist who may imagine either that the mechanical action of the integral parts of the orgasm in a fœtal state accounts for the development of life; or that certain affinities, in the gradual formation of the tissues, have, by virtue of electro-chemical action, developed life; or, entertaining mystical notions respecting material and spiritual agency, he may refer the principle of life to the soul itself. Thus the complexity of the structure of the higher order of animals has led to a great variety of hypotheses; but if we confine ourselves to the simplest organic condition under which life manifests itself, we discover no complicated structure which can suggest either a mechanical, chemical, or psychical cause for its developments.

Accordingly, our own observations and reflections induce us to believe in the existence of a *principle* of life (and we know no better word to designate our meaning) independent of these various organic condi-

* Ehrenberg on the Origin of Organic Matter, from Simple Perceptible Matter, *apud* Taylor's Collection of Foreign Scientific Memoirs, vol. i. p. 566.

tions, and antecedent to all structure, being itself creative, and presiding over the formation and determining the rudimentary development of all beings; and we furthermore believe, that this principle of life, by its own determinate laws, preserves, in the process of organization, the definite form of different species, of animals and plants. Hence arises the uniformity of type observable in the animal creation; hence the unity of organization observed by Blumenbach, upon which he founded his doctrine of the nisus formativus. We cannot, indeed, better explain our meaning than by citing the words of Geoffrey St. Hilaire, who fully recognised the facts, although not the theory we now advocate:—"Par cette expression" (the principle of life to which we apply these words, but which Geoffrey St. Hilaire applied to the nisus formativus of Blumenbach) "on comprend les efforts ou la tendance de l'organization pour se développer, d'une seule et même manière, pour donner les résultats que nous disons ceux de la règle pour faire réapparoitre des produits qui répètent exactement les formes des anciennes races."* Hence, when life is transmitted from parent to offspring, the ovum having received the principle of life which is initiative to the change of its condition, begins gradually to develop a new form, which ends in the organization of another individualism. The constant tendency of the vital laws to preserve uniformity of type becomes very remarkable in certain animals which do not require some organs which are common to their species, and which organs, therefore, do not advance in their development, but are left in a rudimentary state. The whale in embryo possesses the rudiments of teeth, but these not being required, the soft fanlike baleen appears in their stead. So also certain organs, the functions of which are required in one sex and not in the other, are fully developed only in the one which needs them, but stop short and remain in a rudimentary state in the other. In marsupial animals (the kangaroo) the female of the tribe has a process of bone proceeding from the pubes to support the pouch; but the male marsupial having no pouch, the bone appears only imperfectly developed. In the human race the mammæ are necessary for the female to nourish her offspring; but the type is preserved—they exist in a rudimentary state also in the male. We cannot conceive this unity of type to exist unless we ascribe to the principle of life certain determinate laws which modify the development of organization.

All the phenomena of life with which we are conversant support this view; thus, the principle of life being independent of the organic structure in which it exists, requires certain external conditions—such as the presence of light, moisture, and a given temperature for its development.

* Mems. du Muséum d'Histoire Naturelle, 9e année, p. 203.

The vitality of vegetable seeds, and the eggs of animals, will remain latent for an indefinite period. We are informed by Professor Lindley that he has raised three raspberry plants from seeds taken out of the stomach of a man whose skeleton was found in a barrow near Dorchester, thirty feet below the surface of the earth. His body had been buried with some coins of the Emperor Hadrian, and there could be no doubt but that the seeds were 1600 or 1700 years old. There are many instances of the germination of grains of wheat found enclosed in the wrappers of Egyptian mummies. The desiccation of insects will also suspend for years the manifestation of life. The microscopic wheel animal, and the eel (*vibrio tritici*), which causes the ear cockle in wheat, will continue for twenty or thirty years in a dry and apparently dead state, but exposure to air and moisture quickly revives them. The most simple infusoria and rotaria become torpid upon being deprived of moisture; so also will the common garden snail, if put in a dry place, but it immediately revives upon application of moisture. The phenomenon of *hybernation* is also directly connected with the principle of life; it is indeed observed in those animals which become torpid during the winter season, that when the sensibility of all the functions is lessened,—the temperature lowered, the circulation slower, respiration imperceptible, and digestion suspended, vitality becomes more tenacious than ever under these physical abnormal conditions.* Fish and cold-blooded animals survive an intense torpidity: "The fish froze," says Captain Franklin, "as fast as they were taken out of the nets, and in a short time became a solid mass of ice, and by a blow or two of the hatchet were easily split open, when the intestines might be removed in a lump, and if in this completely frozen state they were thawed before the fire, they recovered their animation." The persistency of vitality, even after mutilation of the body, is in most of the cold-blooded animals remarkable, and the restoration of lost parts opens before us a still wider field for observation.

Here also we may refer to a curious fact observed by physicians—viz., the transference of vitality which appears to take place when young persons are habitually placed in contact with the aged. This is not a nursery fiction. It is well attested by very competent authorities. "A not uncommon cause," observes Dr. James Copland, "of depressed vital power is the young sleeping with the aged. This fact, however explained, has been long remarked, and is well known to every unprejudiced observer. I have on several occasions met with the counterpart of the following case—I was, a few years ago, consulted about a pale sickly, and thin boy, of about four or five years of age. He appeared to

* Elliotson's Human Physiology, p. 698.

have no specific ailment, but there was a slow and remarkable decline of flesh and strength, and of the energy of the functions—what his mother very aptly termed a gradual blight. After inquiry into the history of the case, it came out that he had been a very robust and plethoric child up to his third year, when his grandmother, a very aged person, took him to sleep with her; that he soon afterwards lost his good looks, and that he had continued to decline progressively ever since, notwithstanding medical treatment. I directed him to sleep apart from the aged parent, and prescribed gentle tonics, change of air, &c. The recovery was rapid; but it is not in children only that debility is induced by this mode of abstracting vital power. Young females married to very old men suffer in a similar manner, although seldom to so great an extent; and instances have come to my knowledge where they have suspected the cause of their debilitated state. These facts are often well known to the aged themselves, who consider the indulgence favourable to longevity, and thereby illustrate the selfishness which, in some persons, increases with their years."* Every medical practitioner is well aware of the fact, and parents generally are advised not to allow their infants to sleep with aged persons. Now, it is evident, that if this emaciation arise from the abstraction of the vital power, as Dr. Copland expresses it, the principle of vitality must have an independent existence. But here the question suggests itself, where—supposing this view of the theory of life to be adopted—shall we expect to find the seat of the vital principle? The answer to us is obvious. We should expect to find it diffused throughout every organ and every tissue and distant part of the body, sustaining with unity of effect the functions of all the different organs for the support of the entire system. And were we pressed still further to hazard a speculation as to the material medium through which it becomes so diffused, we should be inclined, both from physiological and pathological evidence, to adopt, with certain modifications, the Hunterian hypothesis, —that the blood is its ostensible site, and vehicle; in confirmation of which opinion it may be observed, that those tissues possess the highest vitality which are endowed with the highest vascularity; and those tissues which are the least vascular exhibit the lowest signs of vitality, such as the appendages to the dermoid system, the hair, nails, &c. Hence, also, if the supply of blood to any part be cut off by ligature, that part loses its vitality, and recovers it again when the circulation is restored.

We have thus seen that the principle of life opposes an active resistance (as is stated in the definition of Bichat) to those physical causes which may tend to disturb or destroy organic functions, and

* Dictionary of Practical Medicine. By James Copland, M.D., F.R.S. Article "Debility," vol. i. p. 75.

thereby acts in accordance with a law peculiar to itself. If this be admitted, and we cannot conceive it doubted, we must, by a legitimate deduction, come to the conclusion that such a principle exists. It is indeed obvious that the first effect of extreme cold is upon the vitality of the system, and then supervene loss of power in the capillary vessels, and a diminution and gradual abolition of sensibility. So also, during the prevalence of an epidemic disease, the infected atmosphere strikes upon the life principle itself, and produces that peculiar depression of feeling which is the premonitory symptom of the attack. Again: instances occur of the sudden and peremptory extinction of vitality, by shocks of electricity, the communication of unexpected calamities, and even by a severe blow; and often in these cases, upon *post mortem* examination, no lesion of structure or pathological appearance of any kind can be detected. Here, then, the principle of life is extinguished, leaving the organization from which it was supposed to have originated, absolutely intact. We, therefore, come to the conclusion, from these and other observations, that life is an antecedent principle of organism, derived originally from the Creator, and transmitted through successive generations, whether by germs or ova throughout the infinite varieties of species which exist in the vegetable and animal kingdoms, from parent to offspring, governing by its own plastic laws the forms which it developes; and we, furthermore, consider that this principle of life is not to be confounded with the mind or with the soul, but believe them to be three separate entities, the distinctions between which are capable of being clearly defined.

